# Supplementary material for: Butyrate modulates mucin secretion and bacterial adherence in LoVo cells via MAPK signaling
Source: PLoS One. 2022 Jul 14;17(7):e0269872. doi: 10.1371/journal.pone.0269872 (PMC9282476; doi:10.1371/journal.pone.0269872)
Supplement: Attachment — Submitted filename: Response to Reviewers.docx [file pone.0269872.s001.docx]

Response to reviewers

Thank you for your review.

Our researchers did their best to make modifications based on the reviewers' responses.

1. Page 6, Line 115-125, ELISA kit to measure mucin section: what type of mucin does this kit target? What type of antibody is used in the kit? What is Reagent A and Reagent B?

The contents of the ELISA kit were added to the manuscript (page 6, line 116-124)

2. Page6, Line 132, please specify “primary antibody”. Line 133, what type of secondary antibody used here? Please specify.

- Type of the antibodies was mentioned in the manuscript (page 6, line 134-135).

3. The authors did not clearly specify the mucin they measured is mucins stored in the cells or the mucins that were released (secreted) outside the cell. In another word, is butyrate able to increase mucin synthesis or secretion, or synthesis and secretion? The protocols provided in the manuscript seems not clear about this.

- We were measured to the mucins stored in the cells (page 6, line 114)

4. Does butyrate change the mucin types synthesize by the cells? Many types of mucins are produced by GI epithelial cells, they have different functions and properties.

- There were reports that butyrate stimulates the synthesis or secretion of mucin in the colon cell lines. However, the mechanism by which intestinal epithelial cells increase mucin synthesis or secretion by butyrate treatment needs to be further studied. (page 13, 265-267).
