# Supplementary material for: Butyrate modulates mucin secretion and bacterial adherence in LoVo cells via MAPK signaling
Source: PLoS One. 2022 Jul 14;17(7):e0269872. doi: 10.1371/journal.pone.0269872 (PMC9282476; doi:10.1371/journal.pone.0269872)
Supplement: Attachment — Submitted filename: Response to Reviewers.docx [file pone.0269872.s003.docx]

Response to reviewers

Dear reviewers and editorial staffs in PLOS ONE

We are sincerely grateful for your thorough consideration and scrutiny of our manuscript, “**Butyrate modulates mucin secretion and bacterial adherence in LoVo cells via MAPK signaling**”, manuscript number PONE-D-21-40151R2. Through the accurate comments made by the reviewer, we better understand the critical issues in this paper. We have revised the manuscript according to the Reviewer’s suggestions. We hope that our revised manuscript will be considered and accepted for publication in the PLOS ONE. We acknowledge that the scientific and clinical quality of our manuscript was improved by the scrutinizing efforts of the reviewers and editors. Our researchers did their best to make modifications based on the reviewers' responses and point-by-point responses to the reviewer’ comments are provided below.

**Reviewer**

**1) Reviewer’s comment:** (Page 6, Line 115-125) ELISA kit to measure mucin section: what type of mucin does this kit target? What type of antibody is used in the kit? What is Reagent A and Reagent B?

**Author’s response:** The ELISA kit used in the study targets mucin2. The antibodies used in the kit are capture antibody and biotin-conjugated detection antibody specific to mucin2. The protocol of the ELISA kit included information of reagent A and B were revised in the manuscript (page 6, line 115-130).

**2) Reviewer’s comment:** (Page6, Line 132) please specify “primary antibody”. Line 133, what type of secondary antibody used here? Please specify.

**Author’s response:** Primary antibody and a biotinylated secondary andibody used specific to mucin2 (page 6, line 136-137).

**3) Reviewer’s comment:** The authors did not clearly specify the mucin they measured is mucins stored in the cells or the mucins that were released (secreted) outside the cell. In another word, is butyrate able to increase mucin synthesis or secretion, or synthesis and secretion? The protocols provided in the manuscript seems not clear about this.

**Author’s response:** We measured the mucin2 that secreted outside the LoVo cells to investigate stimulated by the butyrate treatment in the LoVo cells. We found that in this study, butyrate addition can stimulate the secretion of mucin in LoVo cell, and the protocol has been modified more clearly (page 6, line 115-121).

**4) Reviewer’s comment:** Does butyrate change the mucin types synthesize by the cells? Many types of mucins are produced by GI epithelial cells, they have different functions and properties.

**Author’s response:** We think that butyrate may increase mucin produced by cells. However, I would appreciate your understanding that this present study has a limitation of not investigating all types of mucins that can increase in the cell by the butyrate treatment.

Although the underlying molecular mechanism of increased mucin secretion by butyrate treatment remains to be determined, the results of this present study indicate that butyrate may stimulate secretion of mucin in the LoVo cells. Other papers have been also reported that butyrate stimulates the secretion of mucin in the colon cell lines (page 13, line 265-273).

**5) Reviewer’s comment:** The authors did not provide any information I requested, such as what is the antibody target? MUC2, MUC5AC, MUC6, MUC7, MUC8, MUC9, MUC20. The issue about mixing up the synthesis and secretion (release) is not resolved in the revised manuscript.

**Author’s response:** In this study, ELISA kit and immunohistochemical staining kit were used to measure MUC2, and the antibodies also used target for MUC2. More detailed protocols have been modified in the manuscript (page 6, line 115-124; page 6-7, line 136-138)

The purpose of this study is to investigate the effect of butyrate on mucin released from Lovo cells, and we focused on measuring mucin released from butyrate-treated LoVo cells. We expression for synthesis was deleted and the protocol was more clearly modified so that the secretion and synthesis were not confused in the manuscript (page 6, line 115-121; page 10, line 210-213; page 271-273)

I would appreciate your understanding if there is anything insufficient about the author's response and please give us more detailed comments.
